# The organization of Danish emergency departments may not have allowed for a full realization of their performance potential

**DOI:** 10.1186/1757-7241-23-S1-A52

**Published:** 2015-07-16

**Authors:** Anders Møllekær, Iben Duvald, Børge Obel, Jacob Eskildsen, Hans Kirkegaard

**Affiliations:** 1 Center for Emergency Medicine, Department of Clinical Medicine, Aarhus University & Aarhus University Hospital, Aarhus, Denmark; 2 Interdisciplinary Center for Organizational Architecture (ICOA), Department of Social and Business Science, Aarhus University, Aarhus, Dnmark

## Background

According to The Danish Medical Association the 21 Danish emergency departments (EDs) have different organizational designs. Research shows that a 30% performance loss (i.e. quality of care and efficiency loss) can be attributed to organizational design. The aim of this study is to investigate the organizational design of Danish EDs and point to where the full potential of the EDs may not have been reached.

## Method

The study uses a qualitative design. Eight hospitals participated in the study. At each hospital five-six recorded semi-structured interviews were conducted with hospital management, ED leaders, physicians, nurses, and secretaries. Data on the ED's organizational design were collected and analyzed using the multidimensional contingency model. The model describes the relationship between the building blocks that constitutes any organization: Organizational scope, strategy, environment, configuration and complexity, knowledge exchange, process and people and coordination, and control. Alignment between the building blocks is the ideal state (fit). Misalignment (misfit) is a state that is likely to cause decreased performance. The interviews took place from October to November 2013 and from May to August 2014.

## Results

Two main designs were identified: A functional design and a process-orientated design. Both shared the same goal, strategy, and environment. The functional design was organized around the medical and surgical specialties. It had misfits caused by a predominantly functional configuration, ad hoc based communication, limited incentives to do work in the ED, and difficult coordination and control of work processes. The process-orientated design was organized around the patient care process. It had misfits related to staff competencies (people) and coordination and control. In addition, four EDs had a process-orientated design during daytime and a functional design during evening/night time, thus greatly increasing the number of misfits.

## Conclusion

ED organization is very complex. Four out of eight EDs had two organizational designs. There seem to be unrealized potential to improve performance further. Research is needed to expand the study to include the remaining EDs and the relationship between the organizational design and performance.

